# Filling the gaps: Information sources and needs of patients with intestinal failure and their caregivers

**DOI:** 10.1016/j.intf.2026.100385

**Published:** 2026-07-14

**Authors:** Marie L. Neumann, Katie E. Corcoran, Jessica Y. Allen, Swapna R. Kakani

**Affiliations:** a Department of Surgery, Division of Transplant Surgery, University of Nebraska Medical Center, Omaha, NE, USA; b The gutsy perspective, Huntsville, AL, USA; c Department of Sociology and Anthropology, West Virginia University, Morgantown, WV, USA; d Department of Psychology, Howard College of Arts & Sciences, Samford University, Birmingham, AL, USA

**Keywords:** Intestinal failure, Short bowel syndrome, Information access, Information needs, Patient, Caregiver, Survey

## Abstract

**Background:**

Health information-seeking is a central strategy used by patients and caregivers to cope with chronic illness. This study investigated the sources of disease-specific information utilized by adult patients with IF and caregivers of pediatric IF patients to identify existing information needs.

**Materials and methods:**

Using a community-driven research design, we developed a cross-sectional questionnaire to identify information sources and needs. The survey was disseminated via relevant patient support organizations, personal networks, and disease-specific social media support groups. Descriptive and univariate analyses were conducted to identify common information sources and needs and assess differences by respondent type.

**Results:**

A total of 283 respondents, consisting of adult patients with a history of IF (n = 167) and caregivers of pediatric patients with a history of IF (n = 116), completed the survey. Only half were receiving care via an intestinal rehabilitation program. The most frequently used sources of IF-specific information were specialist providers or teams (64%) and social media support groups (52%); a significantly larger share of patients than caregivers reported general practitioners as an information source (30% vs. 13%, p = 0.001). Top information needs pertained to 1) better understanding the current state of the field, 2) optimizing day-to-day life with IF and improving quality of life, and 3) supporting mental health and fostering a sense of connection.

**Conclusion:**

Within this sample of engaged and resource-seeking participants, we found respondents utilized multiple information sources and expressed a range of information needs. We offer several suggestions for addressing these needs.

## Introduction

Patients with intestinal failure (IF) and their family caregivers manage multiple complex and chronic care needs in the home setting, with implications for all areas of life [1], [2], [3]. Health information seeking is a central strategy that individuals impacted by chronic illness use to support coping, inform decision-making, foster confidence and self-management, and manage uncertainty and stress [4], [5], [6], [7]. Access to high-quality, up-to-date health information is particularly critical in the rare disease context, as patients and family caregivers must develop a high degree of health literacy due to widespread knowledge gaps in disease-specific information among healthcare providers and the general public [8], [9]. To meet their need for disease-specific information and support, rare disease patients and caregivers consult informal resources, including online and peer-to-peer modalities [9], [10], [11], [12], more frequently than any other patient population [13]. While the health information-seeking behaviors of IF patients and their families have not been extensively studied, some studies suggest this population may have significant unmet information needs [14], [15], [16]. The purpose of this study was to investigate the information needs and information-seeking behaviors of IF patients and caregivers and propose evidence-based strategies aimed at addressing existing information gaps.

## Materials and methods

### Study design

This project utilized a community-driven research approach that centered patients and caregivers as full partners and initiators across the research continuum. Members of the study team, who are themselves IF patients or caregivers, designed a cross-sectional survey through discussion and consensus; an additional IF caregiver community stakeholder and an adult IF clinician provided instrumental feedback. The aim of the questionnaire was to explore the needs, priorities, and health information-seeking behaviors of patients with IF and caregivers. The instrument was tested for readability and content and face validity; minor adjustments to the wording and length were made in response to feedback. The readability of the final questionnaire was assessed at a Flesch-Kincaid grade level of 9.2. The survey (Supplement) contained 39 closed- and 5 open-ended items that solicited data on sociodemographic and medical background, sources of IF-related information, and existing information needs. It also included multiple items to assess the use of social media to access health information and support, as well as patient/caregiver needs more generally. For conciseness, the latter were not included in the findings presented here. The final questionnaire was uploaded to the REDCap electronic data capture system.

The survey was disseminated directly to the IF community via multiple mechanisms. Firstly, it was shared via email blast and social media posts by *the gutsy perspective*, a community-driven research inititative co-founded by members of the study team (MN, SK, and JA). It was also disseminated via email and social media by relevant patient support organizations (e.g., the Oley Foundation, Intestinal Rehab & Transplant Unwrapped, and the International Foundation for Gastrointestinal Disorders). Thirdly, a search was conducted to identify relevant disease- (e.g., short bowel syndrome, Hirschsprung’s disease) and therapy- (e.g., parenteral nutrition, tube feeds) specific Facebook support groups for patients or caregivers. The authors (MN and SK) were already established members of groups, or when appropriate, attempted to join by contacting the group administrator(s) to describe the purpose of the study and request assistance with disseminating the survey. In some cases, the survey was posted directly by the study team (MN and SK); in other cases, it was posted by the group administrator for greater visibility. When possible, the questionnaire was reposted within 1–2 weeks. Lastly, members of the study team shared the survey via their personal social media profiles and personal communication channels. Data collection took place from October to December 2022 and was covered under an existing IRB protocol (approval 079–21-EX).

### Study participants and setting

Adult (ages 19 +) patients with a history of IF or adult caregivers of patients with a history of IF of any age were eligible to participate in this study. Those who did not meet these criteria were excluded, along with eligible respondents who did not complete the survey. No restrictions on participation were applied by geographic region of residence. All participants electronically provided informed consent, including consent to publish, on the survey’s landing page before advancing to the questionnaire.

### Statistical analyses

Due to small sample sizes for caregivers of adult patients (n = 24 parents of adult patients and n = 8 caregivers of partners) and expected differences in information sources and needs between caregivers of pediatric and adult patients, we excluded caregivers of adult patients from the analyses, along with caregivers who identified their relationship to the patient as “other” (n = 5). Descriptive statistical analyses were conducted to summarize select survey data. Means were calculated for age at diagnosis and years since diagnosis. In addition to calculating the share of respondents who endorsed each item as an information need, an additive index representing a count of topics participants desired information about was calculated. Medians were calculated for the additive index, and percentages were calculated for categorical measures. Medians or proportions for caregivers of pediatric patients versus adult patients were compared using the Wilcoxon rank sum test for medians due to non-normally distributed data and two-sample z-tests for proportions. Statistical analyses were performed using Stata/MP 14.2 software.

## Results

### Participant demographics

Table 1 presents participant and patient socio-demographic and clinical characteristics, both for the sample as a whole and by respondent type (*i.e.,* caregiver of pediatric patient versus adult patient). A total of 283 respondents completed the survey. Adult patients reporting for themselves comprised 59% of the sample; the remaining 41% were caregivers to pediatric patients with IF. The average respondent was non-Hispanic white, college-educated, and female. Patient respondents were, on average, 50 years old, whereas the caregivers of pediatric patients who completed the survey were significantly younger (*M =* 39 years). The mean age at IF diagnosis was under one year for pediatric patients, and approximately 37.5 years for adult patients. Adult patients were also further out from their diagnosis (*M* = 13 years vs. 4 years post diagnosis). Most patients (78%) were diagnosed with short bowel syndrome (SBS) specifically; approximately 64% of patients were receiving home parenteral nutrition (HPN), and 28% had a stoma or mucous fistula at the time of the questionnaire. Compared with adult patients, a greater share of pediatric patients was currently receiving enteral nutrition (EN) (62% vs. 14%). Notably, as a highly unique characteristic of this study sample, only 50% of patients were receiving IF-related care via an intestinal rehabilitation program (IRP), with notable differences in IRP care by patient group (31% of adult patients vs. 78% of pediatric patients).

**Table 1:** Descriptive Statistics, Overall and by Caregiver vs. Adult Patient.

	**Overall**		**Pediatric**		**Adult**	
	n	Mean or %	n	Mean or %	n	Mean or %
**Pediatric vs. Adult patient**						
Pediatric patient	116	40.99%				
Adult patient	167	59.01%				
**Participant Age**	283	45.18	116	38.85	167	49.57
**Participant Gender**						
Male	28	10%	5	4.31%	23	14.02%
Female	247	88.21%	109	93.97%	138	84.15%
Non-binary	4	1.43%	1	0.86%	3	1.83%
Prefer to self-describe	1	0.36%	1	0.86%	0	0
**Participant Education**						
12th grade or less	5	1.78%	2	1.74%	3	1.81%
Graduated HS/GED	22	7.83%	8	6.96%	14	8.43%
Completed trade school	10	3.56%	3	2.61%	7	4.22%
Completed some college	64	22.78%	19	16.52%	45	27.11%
Received Bachelors degree	98	34.88%	45	39.13%	53	31.93%
Received advanced degree	75	26.69%	36	31.30%	39	23.49%
Other	7	2.49%	2	1.74%	5	3.01%
**Participant Race and Ethnicity**						
American Indian/Alaska Native	2	0.71%	1	0.87%	1	0.60%
Asian	5	1.78%	3	2.61%	2	1.20%
Black or African American	4	1.42%	2	1.74%	2	1.20%
Hispanic or Latino	15	5.34%	11	9.57%	4	2.41%
Native Hawaiian or Pacific Islander	0	0	0	0	0	0
White	249	88.61%	97	84.35%	152	91.57%
Multi-racial	6	2.14%	1	0.87%	5	3.01%
**How respondent heard about survey**						
Personal contact	27	9.54%	10	8.62%	17	10.18%
Support organization	48	16.96%	12	10.34%	36	21.56%
Facebook group	204	72.08%	94	81.03%	110	65.87%
Website	2	0.71%	0	0	2	1.20%
Other	2	0.71%	0	0	2	1.20%
**Patient SBS Diagnosis**						
Patient has IF but not SBS	61	21.71%	22	19.13%	39	23.49%
Patient diagnosed with SBS specifically	218	77.58%	91	79.13%	127	76.51%
Not sure	2	0.71%	2	1.74%	0	0
**Patient IRP Status**						
Yes	142	50.35%	90	77.59%	52	31.33%
No	124	43.97%	20	17.24%	104	62.65%
I'm not sure	16	5.67%	6	5.17%	10	6.02%
**Enteral feeds**						
Never used	97	36.19%	16	14.04%	81	52.60%
Currently use	93	34.70%	71	62.28%	22	14.29%
Previously used	70	26.12%	24	21.05%	46	29.87%
Not sure	8	2.99%	3	2.63%	5	3.25%
**PN/IF hydration**						
Never	8	2.87%	0	0	8	4.91%
Currently use	179	64.16%	81	69.83%	98	60.12%
Previously used	90	32.26%	35	30.17%	55	33.74%
Not sure	2	0.72%	0	0	2	1.23%
**Feeding Tube**						
Never	89	33.21%	8	6.96%	81	52.94%
Currently	120	44.78%	92	80%	28	18.30%
Previously	58	21.64%	14	12.17%	44	28.76%
Not sure	1	0.37%	1	0.87%	0	0
**Ostomy**						
Never	110	41.04%	32	27.83%	78	50.98%
Currently	74	27.61%	30	26.09%	44	28.76%
Previously	79	29.48%	52	45.22%	27	17.65%
Not sure	5	1.87%	1	0.87%	4	2.61%
**Patient Age at diagnosis**	274	22.08	116	0.98	158	37.58
**Years since diagnosis**	273	8.96	115	3.96	158	12.61

### Sources of IF-specific information

Table 2 displays sources of health information frequently utilized by adult patients and caregivers of pediatric patients. Overall, specialist clinicians or medical teams (64%) and social media support groups (52%) were the most frequently accessed sources of IF-specific information. On the other hand, conferences (8%) and blogs (11%) were reported as the least frequently accessed. Compared with adult patients, a larger share of caregivers to children with IF reported frequently accessing IF-related information through specialist providers/medical teams (82% vs. 52%, p < 0.001), social media groups (61% vs. 46%, p < 0.05), and direct contact with peers (35% vs. 22%, p < 0.001). A higher percentage of adult patients reported frequently accessing IF-related information through a general practitioner/pediatrician compared to caregivers (30% vs. 13%, p < 0.01) (Table 3).

**Table 2:** Sources of IF-Specific Health Information, Overall and by Caregivers of Pediatric Patients vs. Adult Patients.

**Where Access Information**	**Overall**		**Caregivers of Pediatric Patients**		**Adult Patients**		**Comparing Pediatric vs. Adult**
	**Total n**	**Freq. (%**^**a**^**)**	**Total n**	**Freq. (%**^a^**)**	**Total n**	**Freq. (%**^a^**)**	**p-value**
Specialist clinician/medical team	281	181 (64.41)	115	94 (81.74)	166	87 (52.41)	< 0.000
Social media support groups	278	145 (52.16)	115	70 (60.87)	163	75 (46.01)	0.015
Internet	280	104 (37.14)	115	38 (33.04)	165	66 (40)	0.236
Scientific articles	278	92 (33.09)	114	38 (33.33)	164	54 (32.93)	0.944
Direct contact with peers	279	88 (31.54)	115	52 (45.21)	164	36 (21.95)	< 0.000
GP/pediatrician	278	64 (23.02)	115	15 (13.04)	163	49 (30.06)	0.001
Home health	275	62 (22.55)	113	29 (25.66)	162	33 (20.37)	0.301
Support orgs	278	59 (21.22)	113	21 (18.58)	165	38 (23.03)	0.373
Blogs	277	31 (11.19)	114	8 (7.02)	163	23 (14.11)	0.065
Conferences	276	23 (8.33)	114	9 (7.89)	162	14 (8.64)	0.825

a Percent who often/very often access information from these sources.

**Table 3:** Information Needs, Overall and by Caregivers of Pediatric Patients vs. Adult Patients.

**Item**	**Overall (n = 283)**	**Caregivers of Pediatric Patients (n = 116)**	**Adult Patients (n = 167)**	**Comparing Pediatric vs. Adult (n = 283)**
	**Freq. (%) or Median**	**Freq. (%) or Median**	**Freq. (%) or Median**	**p-value**
Additive index of the following types of information selected	8	10.00	8.00	0.007
Long-term outcomes	181 (63.96)	84 (72.41)	97 (58.08)	0.014
Quality of life	171 (60.42)	73 (62.93)	98 (58.68)	0.472
Tools/tricks/activities of daily living	158 (55.83)	69 (59.48)	89 (53.29)	0.302
SBS/IF-related diet recommendations	156 (55.12)	81 (69.83)	75 (44.91)	< 0.000
Medications used to treat SBS/IF	151 (53.36)	66 (56.90)	85 (50.90)	0.320
Access to and understanding research related to SBS/IF	145 (51.24)	64 (55.17)	81 (48.50)	0.270
Drug development/clinical trials	144 (50.88)	71 (61.21)	73 (43.71)	0.004
Mental health	135 (47.70)	63 (54.31)	72 (43.11)	0.064
Where to find providers that are experienced with SBS/IF	126 (44.52)	37 (31.90)	89 (53.29)	< 0.000
Hydration products/techniques	125 (44.17)	52 (44.83)	73 (43.71)	0.853
Other patient/caregiver stories	117 (41.34)	60 (51.72)	57 (34.13)	0.003
Drawbacks and benefits of long-term IV nutrition (TPN)	111 (39.22)	46 (39.66)	65 (38.92)	0.901
Social support resources	109 (38.52)	53 (45.69)	56 (33.53)	0.039
Relevant books/blogs/podcasts	105 (37.10)	50 (43.10)	55 (32.93)	0.082
Types of surgeries available and their outcomes	95 (33.57)	42 (36.21)	53 (31.74)	0.434
Weaning off TPN	94 (33.22)	63 (54.31)	31 (18.56)	< 0.000
Central line care/infection prevention	91 (32.16)	37 (31.90)	54 (32.34)	0.938
Tools for communication with providers, family, peers	85 (30.03)	38 (32.76)	47 (28.14)	0.405
Transplant	51 (18.02)	18 (15.52)	33 (19.76)	0.361
Ostomies	45 (15.90)	14 (12.07)	31 (18.56)	0.142
EN products	44 (15.55)	30 (25.86)	14 (8.38)	< 0.000
Types of feeding and draining tubes/management of tubes	37 (13.07)	19 (16.38)	18 (10.78)	0.169
Weaning off EN	32 (11.31)	26 (22.41)	6 (3.59)	< 0.000

### Desired information

Table 2 presents the share of respondents, by respondent type, indicating they desire more information about a range of disease-specific topics. A majority of respondents, regardless of respondent type, desired information about quality of life (60%). Most also reported desiring more information about long-term outcomes (64%), though caregivers reported this need to a greater extent than adult patients (72% vs. 58%, p < 0.05). Over half of respondents desired more information on tools, tricks, or activities of daily life, both overall and stratified by patient type. Fifty-one percent of respondents expressed an information need related to access to and understanding of research on IF. Caregivers of pediatric patients were more interested in information about EN and total parenteral nutrition (TPN) and drug development trials than adult patients: weaning off TPN (54% vs. 18.5%, p < 0.001), EN products (26% vs. 8%, p < 0.001), weaning off EN (22% vs. 3.5%, p < 0.001), and drug development/clinical trials (61% vs. 44%, p < 0.01). A significantly larger share of adult patients than caregivers desired more information about where to find experienced SBS/IF providers (53% vs. 32%, p < 0.001).

## Discussion

To cope with and adapt to life with a complex and chronic condition, patients and caregivers seek out health information [4]. The need for such information is especially great in the rare disease context, with patients and caregivers frequently accessing online and peer support to meet information needs [8], [13], [17]. The information-seeking behaviors and needs of individuals living with IF as patients or caregivers have not been fully investigated, although some research hints at significant information gaps [14], [15], [16]. Using a cross-sectional, community-driven research design, we aimed to investigate information sources and needs in the context of IF. Recognizing the various contextual and sociodemographic factors that may shape how adult IF patients and caregivers of pediatric IF patients experience life with the condition, we aimed to assess how these two distinct subpopulations access disease-specific information and identify information needs, with the goal of suggesting evidence-based strategies to address information gaps.

Over 280 adult patients with a history of IF and caregivers to pediatric patients with a history of IF completed the questionnaire. Notably, only half were receiving specialized IRP care at the time of survey completion. To access health information, patients and caregivers reported utilizing two prominent sources. Nearly two-thirds of respondents reported frequently accessing IF-specific information from specialist providers or medical teams, aligning with prior research indicating that clinicians remain an essential, trusted source of information [14], [18], [19]. The second most prevalent source of health information reported in our study was online social media support groups, a finding that is consistent with work in rare disease populations [8], [13].

Research suggests both risks and benefits to using social media for accessing health information. For example, social media use may support coping [20], [21], foster a sense of empowerment [22], and enhance subjective well-being [23]. Conversely, the prevalence of health-related misinformation on social media is a persistent problem [24]. For rare disease patients and caregivers, social media groups function as an important source of learning and support [11], [25], [26], [27], and will likely continue to play an important role in health information seeking. Future research should investigate how IF support groups on social media shape the lived experiences of patients and their families, and whether the knowledge generated and disseminated in these groups can support condition management and foster innovation in our field. Further, given that social media was frequently used as a source of health information in this population, it is important that future research evaluate what health information patients and caregivers seek on social media, how these populations assess its credibility, how they utilize such information, and any potential clinical benefits or consequences.

Survey respondents indicated a desire for more information about a range of topics. One prominent set of information needs pertained to gaining or maintaining an understanding of the state of this highly dynamic field. With continually improving clinical outcomes in IF and recent developments in clinical care and therapies, there may be a significant mismatch between the information readily available to patients and caregivers and the current clinical and research landscape [15]. Our results indicate that the majority of IF patients and caregivers desire access to information about research and development efforts, including information about long-term outcomes, drug development, and clinical trials. At the same time, only a third of respondents reported frequently accessing disease-specific information via published scientific or medical articles. Empirical research suggests that rare disease patients experience paywalled research articles as a barrier to accessing detailed health information [28]. Thus, one potential avenue for addressing this gap is to foster more open-access publishing in our field [29]. Another mechanism to address this information need is the creation, compilation, and intentional dissemination of lay-language summaries of research findings [30], [31], [32]. Additionally, the development of meaningful partnerships in research and development would help to ensure these activities align with patient priorities and needs and foster the successful, targeted dissemination of research findings back to the community [33].

In clinical practice, providers have a unique and meaningful opportunity to direct patients and caregivers toward credible information sources that align with information needs. Recognizing that both the field and patient/caregiver needs for information evolve over time, IRP specialists and general practitioners or pediatricians should periodically revisit information needs and assess understanding. Providers may find it helpful to refer to patients/caregivers to existing tools and resources [34], [35] and support organizations (*e.g.,* The Oley Foundation, The Gutsy Perspective, the International Foundation for Gastrointestinal Disorders (IFFGD), Intestinal Rehab & Transplant Unwrapped, the American Gastroenterological Foundation), as appropriate. Notably, general practitioners and pediatricians serving patients without access to IRPs may play a particularly important role in assessing information needs and directing patients and caregivers toward appropriate support. Prior research suggests general practitioners have limited knowledge of IF [36] and may benefit from additional resources and educational supports as well (*e.g.*, LIFT-ECHO, [37]).

In the years following diagnosis, information needs increasingly center on practical self-management and quality of life, particularly as patients and caregivers outside acute illness phases focus on integrating the condition into daily life [8], [14], [38]. This aligns with a second set of information needs identified by participants in the current study, which centered on resources supporting self-management, including dietary recommendations, medication guidance, and strategies to enhance quality of life. Evidence from prior research on chronic illness demonstrates that information about self-management techniques improves knowledge, symptom control, confidence, and psychosocial outcomes [39]. For individuals with IF, such information is especially salient given the limited availability of IRPs and specialist expertise [36]. IF-specific educational materials or digital resources that promote self-management and address gaps in patients’ practical information needs beyond biomedical factors could be valuable [14], as seen in other fields [40], [41], [42]. Importantly, meaningful stakeholder involvement is critical for maximizing the benefit of such tools [43]. Finally, given the potential for social media support groups to serve as sources of practical information and social support [11], [27], clinicians may consider recommending these as valuable resources to IF patients and caregivers. At the same time, patients and caregivers may benefit from guidance on identifying credible online sources and reminders to consult with providers before implementing any care changes, given the highly individualized care protocols for patients with IF.

A third major theme emerging from this study centers on the desire for information related to social and mental health support among IF patients and caregivers. The impact of IF on psychosocial well-being has been well documented, with prior research reporting elevated rates of stress, anxiety, and depression among IF patients and caregivers [16], [44], [45], [46], [47], [48]. However, access to and use of clinical mental health services is a system-wide problem and remains limited; IF-specific interventions are still in the early stages of development [16], [47], [49]. Notably, nearly half of our study participants expressed a desire for additional information about mental health, suggesting interest in integrating mental health considerations into IF self-management strategies [47], [50]. Given the well-established association between perceived social support and improved mental health outcomes in other disease states [51], [52], [53], addressing social support–related information needs within the IF community is especially relevant. Our findings indicate that more than half of pediatric caregivers and over a quarter of adult patients sought information about peer experiences, while nearly 40% of respondents desired further resources on social support. These results suggest a strong desire for shared narratives and peer connections, which may foster opportunities for individuals to take an active role in self-managing their psychosocial needs [50]. Indeed, in our previous work, we found that members of the IF community readily and actively participate in brief, narrative storytelling as a form of peer knowledge transfer [54]. Collectively, these findings underscore the need for multifaceted approaches that address both psychological well-being and social connectedness information needs, recognizing these domains as integral components of comprehensive IF care.

An additional notable finding concerned differences between adult patients and the caregivers of pediatric patients in the patterns of information sources used and the information needs identified. A much larger share of caregivers than adult patients frequently accessed disease-specific information via specialist providers or teams. On the other hand, nearly 30% of adult patients but only 13% of caregivers reported general practitioners as a frequently-used information source. Adult patients also desired information on finding experienced providers, ranking it among their top three information needs. Together, these findings highlight a known challenge within our field: limited access to established centers of excellence, particularly for adult patients [55]. Research suggests that non-specialist clinicians lack disease-specific knowledge [36], [56], [57], calling into question their ability to meet the complex information needs of IF patients and caregivers. Our findings thus highlight the importance of information dissemination efforts that extend beyond specialized centers by leveraging community organizations, home health providers, key community stakeholders, and other virtual spaces.

A notable strength of this study was its community-driven research design, which enabled our team to draw on existing relationships and networks to distribute the questionnaire directly to the IF community. Because of this approach, we recruited over 280 participants; more than half were not receiving specialized IRP care at the time of the study and thus represent an understudied population. At the same time, we recognize the questionnaire’s dissemination via Facebook may have biased the results, leading to an overrepresentation of highly engaged and digitally connected individuals. Because of this selection bias, study results may overestimate how frequently social media is utilized as a source of disease-specific information; alternatively (or additionally), information needs may have been underestimated in this sample. The generalizability of our study findings may further be limited by the homogeneity of our study sample, which was largely white, female, and college-educated. At the same time, significant heterogeneity in nutrition support status within the study sample may limit generalizability.

An additional limitation is that adult patients were significantly overrepresented in the “no IRP” group, making it difficult to determine whether differences between groups were due to respondent type (*e.g.,* adult patient vs. caregiver of a pediatric patient) or access to specialized care. Lastly, our questionnaire did not assess factors that can influence information-seeking, such as health literacy, family support, comfort with technology, access to broadband, mental health status, or prior experiences with hospitals/providers/social media [18], [58], [59]. Future research should seek to identify how these factors, as well as disease- or therapy-specific considerations (e.g., IRP status, years since diagnosis), shape the information-seeking behaviors and preferences of IF patients and caregivers.

## Conclusions

Health information-seeking is a central strategy for coping with illness. Using a community-driven, cross-sectional research design, we found that adult patients with IF and caregivers of children with IF rely on specialist clinicians and social media support groups to access disease-specific information. Further, we identified a range of information needs pertaining to better understanding the state of the field, receiving practical information to help optimize day-to-day living and quality of life, and enhancing mental health and a sense of shared experience. Our findings also point to disparities in information access between adult and pediatric populations and highlight the need for adult patients to access information on finding experienced IF providers. Potential strategies for addressing these needs include improving access to research, developing educational materials that provide practical information, and considering patient and caregiver mental health needs as part of the multidisciplinary care approach. Importantly, meaningful partnerships with patients and caregivers in research and development should be central to efforts to address existing information gaps.

## CRediT authorship contribution statement

**Jessica Y Allen:** Writing – review & editing, Writing – original draft. **Katie E Corcoran:** Writing – review & editing, Writing – original draft, Visualization, Formal analysis. **Marie L Neumann:** Writing – review & editing, Writing – original draft, Methodology, Investigation, Formal analysis, Data curation, Conceptualization. **Swapna R Kakani:** Writing – review & editing, Writing – original draft, Project administration, Methodology, Investigation, Formal analysis, Data curation, Conceptualization.

## Patient/guardian consent for use of patient details

Not applicable as no participant details were disclosed in the manuscript. However, as required by the institution’s IRB, informed consent was obtained from all participating patients/ caregivers.

## Ethical Statement

Data collection was covered under ethics approval 079-21-EX.

## Funding

This work was supported by the J.A.M. Jar Simmons Fund for Intestinal Failure Research.

## Declaration of Competing Interest

The authors declare the following financial interests/personal relationships which may be considered as potential competing interests: Marie L Neumann reports financial support was provided by Zealand Pharma. Marie L Neumann reports financial support was provided by Simmons Fund for Intestinal Failure Research. Marie L Neumann reports a relationship with Takeda Pharmaceuticals USA Inc that includes: consulting or advisory, speaking and lecture fees, and travel reimbursement. Marie L Neumann reports a relationship with VectivBio AG that includes: consulting or advisory. Marie L Neumann reports a relationship with Optum Health Education that includes: speaking and lecture fees and travel reimbursement. Jessica Y. Allen reports a relationship with Takeda Pharmaceuticals USA Inc that includes: consulting or advisory. Jessica Y. Allen reports a relationship with Ironwood Pharmaceuticals Inc that includes: consulting or advisory. Swapna Kakani reports a relationship with Ironwood Pharmaceuticals Inc that includes: consulting or advisory and speaking and lecture fees. Swapna Kakani reports a relationship with Takeda Pharmaceutical Company Limited that includes: consulting or advisory, speaking and lecture fees, and travel reimbursement. Swapna Kakani reports a relationship with B. Braun Medical Inc. that includes: consulting or advisory, speaking and lecture fees, and travel reimbursement. Swapna Kakani reports a relationship with American Gastroenterological Association that includes: consulting or advisory. Swapna Kakani reports a relationship with The Association for Vascular Access that includes: board membership, non-financial support, and travel reimbursement. Swapna Kakani reports a relationship with Center for Innovation & Value Research that includes: consulting or advisory. Swapna Kakani reports a relationship with Patient-Centered Outcomes Research Institute that includes: consulting or advisory and travel reimbursement. Swapna Kakani reports a relationship with MT SINAI HOSP MED CTR that includes: consulting or advisory. The remaining others declare that they have no known competing financial interests or personal relationships that could have appeared to influence the work reported in this paper. VCFBVG.
